# Standardized Risk Analysis Approach Aimed to Evaluate the Last African Swine Fever Eradication Program Performance, in Sardinia

**DOI:** 10.3389/fvets.2019.00299

**Published:** 2019-09-13

**Authors:** Federica Loi, Stefano Cappai, Annamaria Coccollone, Sandro Rolesu

**Affiliations:** Istituto Zooprofilattico Sperimentale della Sardegna - Osservatorio Epidemiologico Veterinario regionale, Cagliari, Italy

**Keywords:** African swine fever, negative binomial regression model, risk analysis, epidemiological cycle, Sardinia, eradication program

## Abstract

From more than 40 years African swine fever (ASF) is endemic in Sardinia. Historically, areas at higher risk are located throughout some inland parts of this island where domestic pigs are still illegally kept in semi-wild conditions, living in contact with the local wild boar population, thereby creating perfect conditions for disease endemicity. A new eradication plan (EP-ASF15-18) has been ongoing for the past 3 years, based on a comprehensive strategy adapted to the local situation and focused on strong actions on domestic pig farms, wild boars (WB), and the third Sardinian typical involved population [illegal free-ranging pigs (FRPs)]. A fundamental aspect of the plan is the classification of pig farms as “controlled” or “certified,” based on clinical, structural, and biosecurity characteristics. The eradication plan also provides for strong action against illegal farms and pig meat marketing channels. In addition, this plan establishes specific control measures for WB hunting and ASF checks. Each control strategy is specifically based on municipality risk level, to focus actions and resources on areas at higher risk of endemic or re-emerging ASF. Thus, precise risk classification is fundamental to this goal. The aim of the present work was to establish an ASF risk index, to provide a summary measure of the risk level in the Sardinian municipalities. This synthetic measure can express the different aspects of a multidimensional phenomenon with a single numerical value, facilitating territorial and temporal comparisons. To this end, retrospective data (years 2011–2018) were used. The ASF risk index is the result of the algorithmic combination of numerical elementary indicators: disease prevalence in the suid populations, WB compliance with EP-ASF15-18, domestic pig compliance with EP-ASF15-18, and presence of FRPs. A negative binomial regression model has been applied and predictors calculated to obtain a risk index for each municipality. The result of the risk analysis was discussed and considered according to expert opinion and consensus. The results of this study, expressed as risk score and classified into five risk levels, can be used to help define actions to be carried out in each Sardinian municipality, according to the risk assessment for the territory.

## Introduction

African swine fever (ASF) is one of the most serious infectious diseases affecting domestic and wild pigs, responsible for serious economic and production losses (1). ASF is caused by a large icosahedral DNA virus (family Asfarviridae, genus *Asfivirus*), and characterized by up to 100% mortality (2). The considerable economic losses caused by the disease are even more serious considering the absence of an effective vaccine (3). The quarantine of the affected area and the slaughter of confirmed and suspected infected and contaminated animals (stamping out) in an outbreak, actually are the available methods of disease control, according to European legislation (Directive 2002/60/EC 27/06/2002). In 1921, Montgomery described the first ASF case in Africa and since then the disease is endemic in the African continent with a complicate sylvatic cycle (4, 5). At the end of the 1980s, several countries in Western Europe experienced ASF that were quickly eradicated. However, after its first notification in 1978, ASF persisted in Sardinia involving dense populations of free-ranging domestic pigs (DPs), with occasional incursions in wild boars (WBs) species (6). Since 2007, the disease has been reported in multiple countries including the Russian Federation, Belgium, Hungary, Bulgaria, Latvia, Moldova, Poland, Romania, Russia, and Ukraine, in both domestic and wild pigs (7, 8). Starting in August 2018, the disease has been spreading and having a considerable impact on the pig population of the Asian continent, primarily in China. It should be noted that China has over 50 per cent of the world's pig population, and continue to report outbreaks to date (9). More recently, ASF notifications have been reported from Mongolia in January 2019, Vietnam in February 2019, Cambodia in March 2019, and Hong Kong (SAR-PRC) in May 2019 (10). Recently, new outbreak in Slovakian backyard has been reported (11).

### Geographical Distribution of ASF in Sardinia From 1978 to the Present

**Figure 1:**
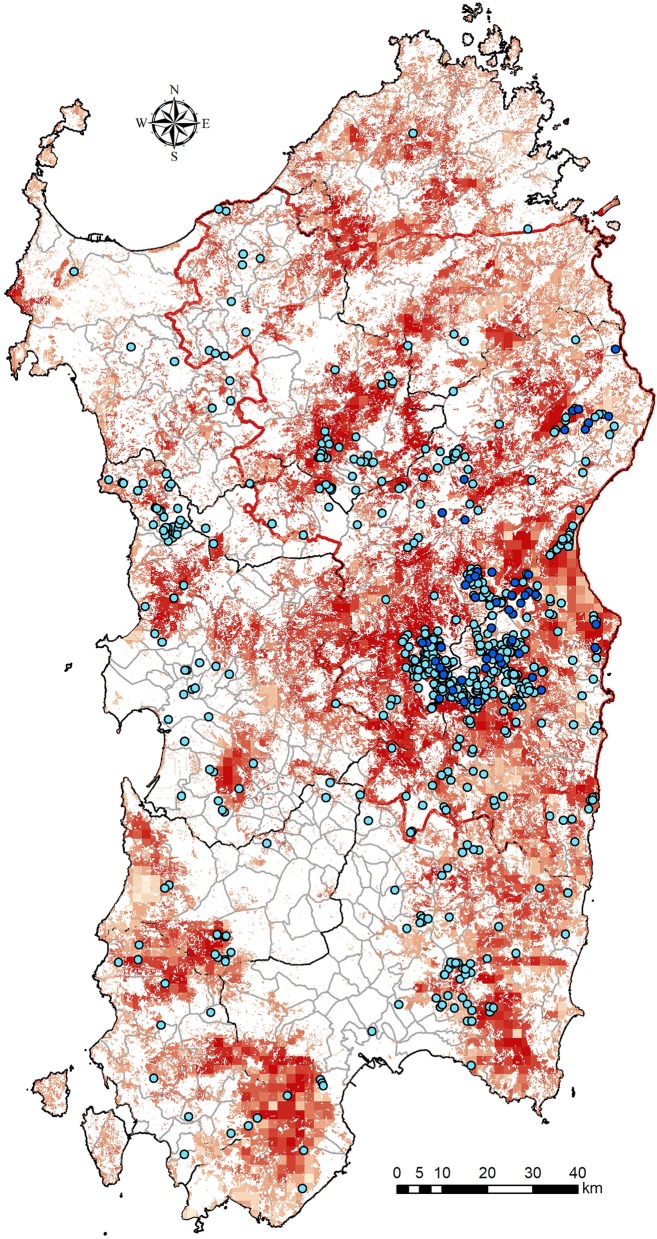
Wild boars density distribution (red squares) in Sardinia and localization of free-ranging pigs (blue dots) during the 2013–2015 years (clear dots) and during 2016–2018 (dark blue dots).

**Figure 2:**
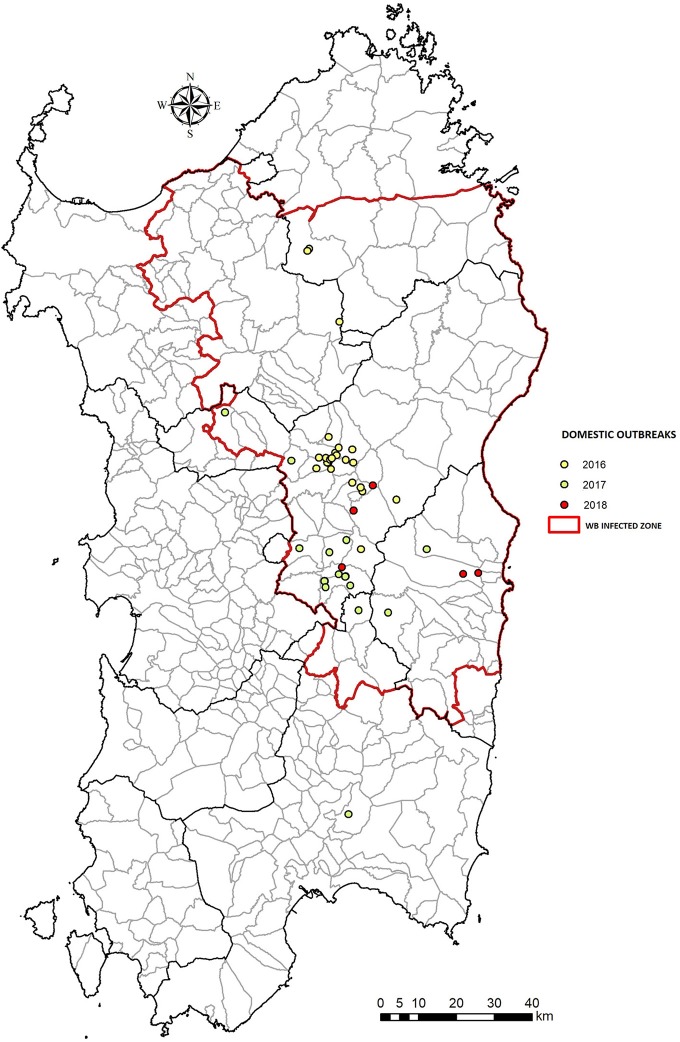
Number of ASF outbreaks in domestic pig farms, between 2016–2018 in Sardinia (Italy). The WB Infected Zone is delimited by the red line.

Forty years have passed since ASF entered Sardinia, probably owing to the upon arrival of processed meat contaminated by African swine fever virus (ASFV) from the Iberian Peninsula (12). The consequence of the first notification of ASF in southern Sardinia (March 1978) was the loss of more than 10,000 pigs. Consequently, serious concerns arose about the difficulties of disease control owing to the specific way that free-ranging pigs (FRPs) were kept in the island's inland areas. The most probable cause for the spread of this disease across the island are the uncontrolled movement of infected pigs which may survive infection, and consequently their introduction into healthy herds and the feeding of waste food containing meat from infected pigs. (13, 14). As soon as the disease spread to central Sardinia (June 1978), it became clear that disease control measures were not being practiced by the local population and that residents had not abandoned local cultural traditions of free-ranging and breeding (15, 16). In addition, the disease spread to the local WB population, creating an even more complex picture. Recently (2015–2018), strict measures have been implemented in Sardinia, aimed at fighting this disease by focusing on hunting management and eliminating illegal FRPs in the latest ASF eradication plan (EP-ASF15-18). The efficacy of this plan is reflected in an evident decrease of disease prevalence over the past 6 years, from 0.61% (95% CI = 0.51–0.74) to 0.007% (95% CI = 0.003–0.1) on DP farms, from 0.32% (95% CI = 0.22–0.46) to 0.04 (95% CI = 0.01–0.09) of ASFV positivity and from 6.23% (95% CI = 5.62–6.89) to 1.12% (95% CI = 0.84–1.49) of seropositivity in the WB population. Detailed spatiotemporal distribution of ASF over the years was provided by Mur et al. and an overall picture of outbreaks from 2011 to 2016 was described by Cappai et al. (17). In Sardinia, the disease is confined to the central part of the region (Figures 1, 2), except for one isolated case near Cagliari in the south (2017), where ancient habits steeped in tradition persist and the disease has become endemic (17–19). No evidence of ASFV has been found in DPs since September 2018.

### Sardinian ASF Epidemiological Cycle

A unique and particular ASF epidemiological cycle has been present in Sardinia since 1978. Both the ticks of the genus *Ornithodorus* and other natural reservoirs, such as the warthog (*Phacochoerus africanus*) (20–22), which constitute the well-established “natural host–vector–pathogen system” or “sylvatic transmission cycle” of ASF (23, 24), are absent in Sardinia. In Sardinia, the disease occurs mainly as a result of interaction between the three suid populations, i.e., DPs, WB, and FRPs. On this island, ASFV is characterized by a more anthropogenic cycle in which FRPs (rather than warthogs) assume the role of epidemiological reservoir and act as the link between the other two suid populations, without the involvement of *Ornithodorus* ticks. The involvement of insect vectors other than *Ornithodorus* in disease transmission has not been excluded and is the object of ongoing studies in Sardinia. The three suid populations involved interact with each other in a more or less intensive manner, depending on the management of pig farms (biosecurity), hunting management, and observance of rules governing animal identification and registration. Given that the spread of ASFV in DPs is facilitated by human activities and animal movement (i.e., live infected animals or contaminated meat and other by-products), as demonstrated in many studies (17, 25–27), the consequent spread of disease is related to the growing human population and increasing number of DPs. Furthermore, human activities are the primary cause of long-distance ASF transmission (28). An exclusive and primary role of WBs in the persistence of this disease on the island has never been recognized (17, 29), and the irrelevant role of WBs in the maintainance of disease endemicity in absence of continuous source of virus has been demonstrated (30). Notwithstanding, the contribution of WBs in ASFV maintenance is owing to contact with the FRP population via live or dead animals (carcasses). As shown in Figure 1, illegal FRPs are distributed throughout high-density areas of WBs; thus, contact between these populations is estimated to be frequent and intensive. In contrast to consolidated active surveillance (i.e., during hunting season), passive surveillance aiming to locate and test WB carcasses is in place on Sardinia. During the past 2 years (2017–2018), a total of 278 WBs (i.e., hunted or found dead) have been collected and tested for ASFV, with dead animals showing similar but slightly lower prevalence than hunted animals (2.1%, 95% CI:). However, a significantly higher prevalence has been detected in FRPs for both seroprevalence 53.4% (95% CI: 50.6–56.3) and virus prevalence (2.6%; 95% CI: 2.1– 3.0) (18). Although these prevalence values have decreased with increased culling actions in the same area, these findings seem to confirm the key role of the FRP population in the persistence ASFV in Sardinia over the past 40 years.

### Role of Illegal Free-Ranging Pigs (FRPs) in Disease Persistence

From the first ASF notification in Sardinia several eradication plans have been put in place at regional level, with special focus on DPs and WBs populations. From the first eradication program in 1982, many others have been carried out, with widely varying results. Some of these were able to came close to the ASF eradication, but none was able to solve the problem presented by FRPs, which in Sardinia have a key role in the spread and persistence of disease (17–19, 31). The breed of few pigs in small backyard is common ancient practice in Sardinia. This manner of keeping pigs in free-ranging conditions is inherent to the cultural traditions of their owners; thus, pig owners refuse to change their habits because this would mean losing their cultural identity (13, 14, 32). The old practice has become a problem when the number of illegal FRPs drastically increased using free common land allocated to agriculture (18). Furthermore, illegal FRPs constantly come into contact with WBs, favoring the spread of disease and hindering its control. The role of FRPs in virus persistence has been previously suggested by many researchers (13, 14, 31); however, this issue has only recently been fully elucidated, thanks to the more stringent measures of EP-ASF15-18 to combat FRPs and any kind of illegal activity in the swine sector (18). These illegal unregistered animals have been defined as a virus reservoir that is out of the control of official channels, acting as a virus link between the other two pig populations: legal pigs kept on backyard farms and WBs. Up to the present, 3,800 FRPs have been culled in various parts of central Sardinia. To date, many studies have contributed to better understanding and quantifying the role of the most common factors involved in the persistence of ASF. However, many issues, such as the role of illegal FRPs and socioeconomic status of pig farmers, remain unclear and need to be studied in depth. In the present work, we aimed to perform a quantitative risk assessment based on all suid populations involved in the endemic persistence of ASF in Sardinia, as well as social factors, which could help to identify farms or municipalities at high risk for ASF occurrence or persistence. The result of this analysis is to create a band risk map in which the ASF risk for each Sardinian municipality has been calculated. On the basis of our results, subsequent actions of the EP-ASF15-18 can be planned and implemented, toward the goal of ASF eradication on Sardinia.

## Materials and Methods

### Study Area

Sardinia island has an average area of 24,000 km^2^, located in the middle of the Mediterranean Sea (40°03′N 9°05′E). The island characterized by various ecosystems, mounts, woodlands, lowlands, largely uninhabited areas, rivers, long sandy beaches, and rocky coasts. Sardinia is administratively divided into 377 municipal territories, covered by eight different Local Socio-Sanitary Areas (ASSL).

**Figure 3:**
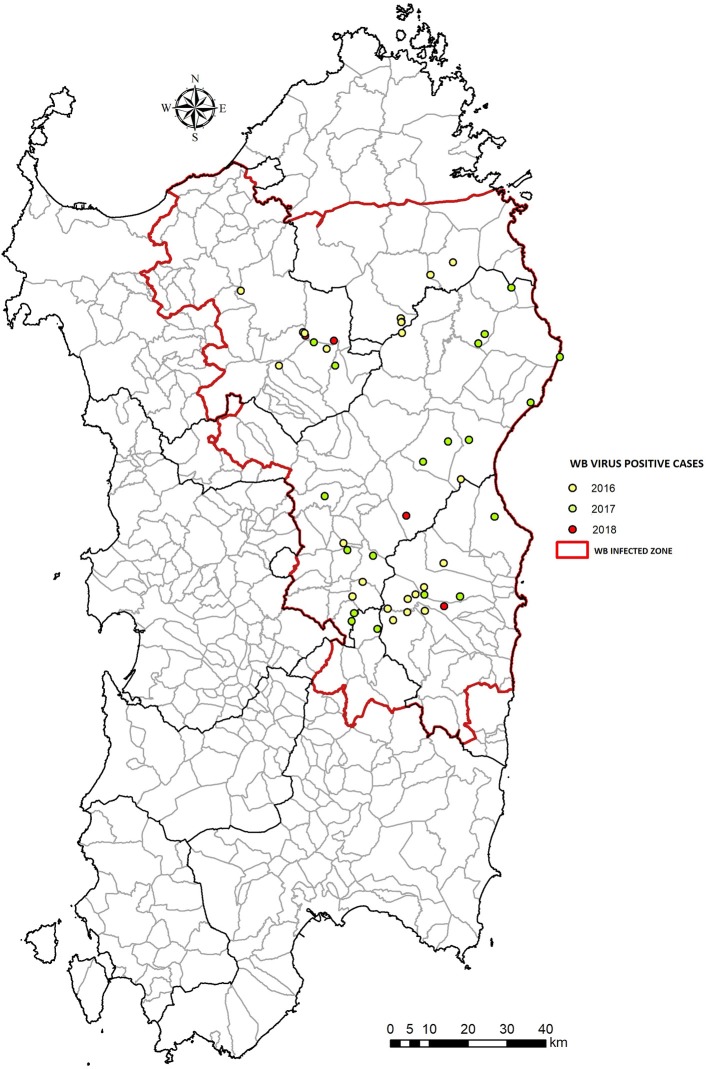
Number of wild boars ASFV positive from 2016 to 2018 in Sardinia (Italy). The WB Infected Zone is delimited by the red line.

The coexistence of a modern economy within a vast unspoilt territory makes Sardinia one of the few examples in Europe of an integrated rural and modern society. Despite the vastness of its territory, Sardinia is characterized by largely uninhabited areas, that make it the third Italian region in terms of population density (33) Given the sparse population (69 inhabitants/km^2^) and the presence of pristine areas, 48% of the island is used for pastoral and agricultural activities; of this proportion, 60% is used for breeding sector, 35% for planting, and the remainder for wood cultivation (34). Although pig livestock in Sardinia dates back to the 6th century BC, swine farming has always been secondary source production, limited to self-consumption. On the other hand, sheep and goat husbandry has always been primary production in Sardinia. Indeed, the culture of breeding one or a few pigs is still a very common practice, mostly in mixed farms where swine and sheeps are commonly breeded (13, 14, 18, 35, 36). As establish by the EP-ASF15-18 (“VI measure concerning the fight against ASF in WB population,” Regional decree n.9, 07/06/2017), an inner Sardinian area of a total of 9,000 km^2^, named “Infected Zone,” has been adopted to apply stronger measure against the disease in sylvatic populations, and includes 121 municipalities (Figures 2, 3).

### Data Collection

This retrospective study covered an 8-year period of analysis (2011–2018) and included data regarding to the three suid populations involved in ASF persistence: DPs, WBs, and FRPs. Each of the 377 Sardinian municipalities was considered as epidemiological unit, arranged by study year and linked to all 59 variables collected (Table S1). For the purposes of this work, all ASF outbreaks occurred in DP farms between 2011 and 2018 constituted the outcome of this study. Based on official data recorded in the Italian National Information System for the Notification of Infectious Animal Disease (SIMAN) database, an ASF outbreak was defined as a diagnosed disease event in DP farm, in accordance with the World Organization for Animal Health (OIE) Manual of Diagnostic Tests (37). Several characteristics of the infected pig farms such as province and municipality, data of suspected diagnosis, data of confirmed diagnosis, and including both extensive and backyard pig farms, were collected. Because the outcome was the number of ASF outbreaks in the year and municipality of reference [considering both seropositive and virus-positive domestic pig farms (SVDPs)], the dependent variable SVDP follows a count data distribution rather than a normal distribution. We conducted an extensive review of the existing proven risk factors for ASF occurrence, to ensure completeness of this study (17–19, 26, 38–40). An *ad-hoc* database was created to collect detailed and complete information from various sources, based on municipality level data. Data related to DP farms (category A) were as follows: the number of SVDPs for ASF; the number of registered and active farms, including those active throughout the year (activity start date January 1 or later and end date not before December 31); the number of pigs, using data from March 31 as this is the date of the official census; data of animal movement (number of animals introduced to/removed from farms from one municipality to another). These data were collected from the official veterinarian databases: the Italian Veterinarian National Database (BDN), Veterinary Information Systems of the Italian Ministry of Health (VETINFO), and SIMAN. All data collected have been verified on the globally official site for animal health disease (https://www.oie.int/en/animal-health-in-the-world/wahis-portal-animal-health-data/), taking into account the possible inconsistencies due to different update time between Italian national database and OIE international database. The number of official veterinarian checks on pig farms was determined, to calculate the percentage of compliance among DPs. From 2015, this measure is largely used in Sardinia to evaluate the performance of DP farms in terms of ASF management (17). This measure is defined as the proportion of farms complying with EP-ASF15-18 regulation over the total number of farms in the same municipality (reported as a percentage) during the previous year of reference, considering that farmers had a minimum of 6 months and a maximum of 1 year to solve nonconformities found during the previous check (17). Using data on confirmed outbreaks from SIMAN, the present work used the following variables to describe the WB population (category B): areas with WBs, estimated number of WBs living in each municipality, number of hunted and conferred WB, number of WBs tested for the presence of ASFV or ASF antibodies, number of WBs positive for ASFV, number of ASF-seropositive WBs, sex (male or female) and age (older or younger than 6 months) of ASFV-positive WBs, percentage of male ASFV-positive WBs (calculated over all ASFV-positive WBs), percentage of young ASFV-positive WBs (calculated over all ASFV-positive WBs), sex (male or female) and age (older or younger than 6 months) of ASF-seropositive WBs, percentage of male ASF-seropositive WBs (calculated over all ASF-seropositive WBs), and percentage of young ASF-seropositive WBs (calculated over all ASF-seropositive WBs). The WB density estimation of the Faunal Vocation Chart of the Sardinian Region performed by Apollonio in 2012 was used to calculated the number of WB for each Sardinian municipality (41). Furthermore, it was necessary to identify those parts of the territory that could support the habitat cycle of these populations and to define macro areas within which there are about 1,000 WBs, according to current EU regulations (2003/422/EC approving an ASF diagnostic manual, Chapter IV(H)). Alternatively, sufficiently separated parts of the territory were distinguished, in which specific WB metapopulations are present. The overlap of these areas with the administrative limits of municipalities makes possible a correct representation of the wild populations per municipality. According to EP-ASF15-18 rules (42), all WBs hunted inside an infected zone should be tested for the presence of ASFV antibodies. Based on this, supposing that 45% of the total estimated number of live WBs are hunted during the hunting season, the percentage of WB compliance inside an infected zone is calculated as the proportion of WBs hunted over the total estimated WBs hunted in the same municipality during the year of reference. However, in Sardinian regions unaffected by ASF, a total of 58 WBs for each area must be serologically tested, upon which calculation of compliance is based. Based on Regional Wildlife Agency reports and data collected from ongoing actions of FRP depopulation, we collected information about the presence/absence of FRPs and their number, the number of culled FRPs and FRPS laboratory-tested for ASF, and virological and serological prevalence of ASF in these populations, to describe the illegal FRP population (category C). Given that recent studies suggest that socioeconomic status of farmers is strictly related to livestock disease risk (17, 31, 32, 38, 43) and a comprehensive and in-depth knowledge of relevant risk factors is basic requirement for disease prevention (44, 45), we collected a large number of covariates (category D) from the Italian Statistician National Institute database, specifically AgriISTAT (46). To describe the actual situation of pig breeding in Sardinia, data for the characteristics of farm owners, such as sex, age, level of education. and type of farm were collected using fiscal codes recorded in the BDN. A series of social indicators, called territorial indicators for development policies (47) were collected at municipality level and included in the present analysis, given their previously demonstrated contribution to describing the risk of ASF in DPs and WBs (32); these indicators included the Material Deprivation Index (MDI), employment rate, cultural demand, micro-criminality index, rate of tourism in low season, proportion of the population at risk of floods, rate of reported thefts and robberies, forest surface, amount of energy produced from renewable sources, and amount of differentiated waste. Areas (in square kilometers) of asphalted road and water bodies were collected from the Regional Geographical Service (Servizio Informativo e cartografico Regionale, Regione Sardegna, 2011) at municipality level, and 216 these were considered as potential covariates.

### Statistical Analyses

**Figure 4:**
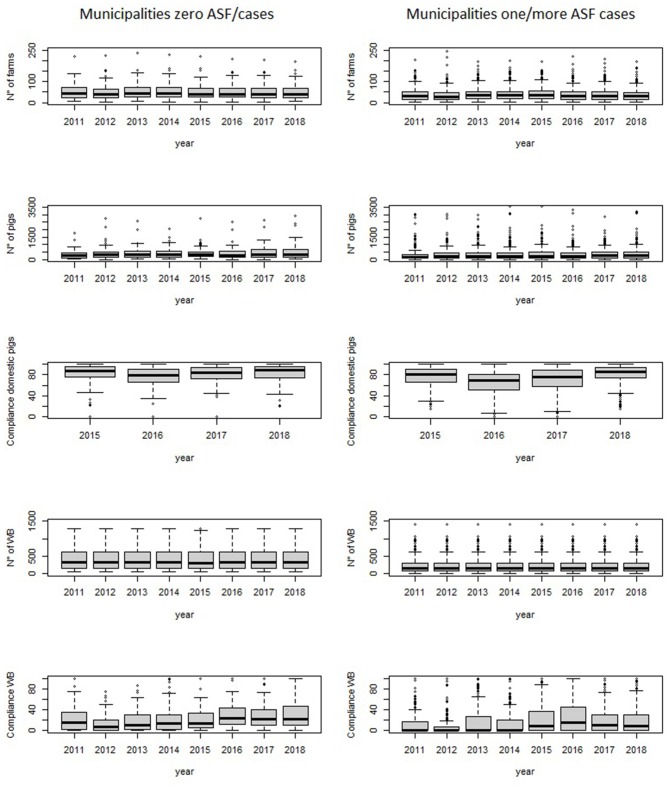
Baseline distribution of the number of farms and number of pigs, domestic pigs compliance with ASF-EP15-18, number of wild boar and compliance with ASF-EP15-18 rules for hunting season management, from 2011 to 2018, according to municipalities with zero ASF cases and one or more cases.

**Figure 5:**
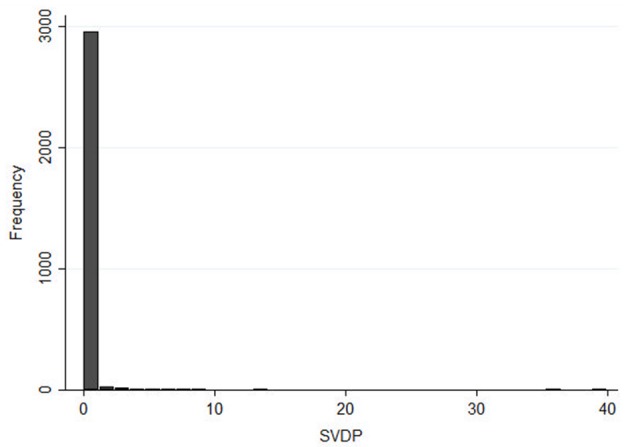
Histogram distribution of NBRM's outcome, number of SVDP in all Sardinian municipalities during the 2011–2018 years.

An *ad-hoc* database has been created using Microsoft Office Access system and all information collected was double blinded and password-protected stored to ensure privacy. Extensive data checking was performed to evaluate the consistency and accuracy of the data collected and any disagreement was analyzed and corrected. Considering epidemiological, experimental, and statistical issues (i.e., non-collinearity), several putative and potentially relevant predictors were detected. The baseline distribution of each explanatory variable was summarized and described, according to municipalities with zero or more/equal to at least one case of ASF (Table 1, Figure 4). Most collected variables were quantitative and expressed as mean and standard deviation (SD) or median and interquartile range (IQR). Frequency (n) and percentage (%) were used to describe categorical data. Considering both experimental and statistical requirements, the features in Table 1 were evaluated as potential covariates in our analyses. The outcome SVDPs represents non-negative count data, and regression techniques cound be used to estimate the mean value distribution. Poisson regression and negative binomial (NB) regression are among the most popular count data regression methods used in epidemiology (48–50). Although Poisson model is suitable for count data with mean equal to its variance, whereas the NB model is more appropriate in condition of overdispersed data with an excessive presence of zero values (51, 52), such as those in our dataset (Figure 5). Thus, the final developed model was a negative binomial regression model (NBRM). This model assumed that the outcome variable Y is the total number of events occurring in a specific space-time interval (here, the number of ASF-positive farms, for both the presence of ASFV or ASF antibodies, in each specific municipality). The earliest definition derived from the binomial distribution characterizes NBRM as the number of failures before the (1/α)th success. Recently, parametrizations have been used to describe the NBRM as derived from a mixture of gamma and Poisson distributions (50, 53). A mixed-effects NBRM was applied (Equation 1), including random effects (year and municipality), assuming not independent observations between years and municipalities and to control this level. Considering a series of M independent clusters, and conditional on the latent variable ζij and a set of random effects *u*j,

$$\begin{array}{l} y_{ij}|\zeta_{ij}\sim Poisson\;\left(\zeta_{ij}\right) \end{array}$$

and

$$\begin{array}{l} \zeta_{ij}|u_{j}\sim Gamma\;\left(r_{ij},p_{ij}\right) \end{array}$$

and

$$\begin{array}{l} u_{j}\sim N\;\left(0,\;\Sigma \right) \end{array}$$

where y_ij_ is the count response of the ith observation, i = 1,…, n_j_, from the jth cluster, j = 1,…, M, and r_ij_ and p_ij_ were parameterized using the mean overdispersion:

$$\begin{array}{l} r_{ij}=\frac{1}{\alpha }\text{ and }p_{\text{ij}}=\frac{1}{1+\alpha \mu_{\text{ij}}} \end{array}$$

**Table 1:** Description at baseline of all variables involved in the African swine fever risk analysis, according to municipalities with zero/one or more cases, related to domestic pigs and wild boars during 2011–2018.

**Variable**	**Municipalities with zero cases**	**Municipalities with one or more cases**
	**(*n* = 2889)**	**(*n* = 127)**
*N* farms	34 [18–55]	55 [33–100]
Pigs censed	240 [121–463]	475 [271–843]
Seropositive farms	0 [0–0]	0 [0–1]
Virus positive farms	0 [0–0]	1 [1–2]
Farms checked	17 [8–32]	25 [11–47]
Movements	159 (77)	265 (190)
Compliance DP	87 [61–96]	80 [63–91]
Estimate living WB	177 [88–353]	494 [259–777]
Estimate hunted WB	80 [40–159]	222 [116–350]
Hunted WB	7 [0–34]	36 [15–61]
Sex WB		
Male	4 [0–11]	7 [1–17]
Female	4 [0–7]	5 [1–9]
Age WB		
< 6 months	3 [0–8]	6 [1–8]
≥ 6 months	3 [0–6]	5 [1–7]
WB virus tested	26 [9–56]	39 [21–50]
WB sero tested	27 [9–58]	38 [15–50]
Virus positive WB	0 [0–0]	0 [0–1]
Seropositive WB	0 [0–0]	4 [0–7]
Virus positive WB_M	0 [0–0]	0 [0–2]
Virus positive WB_F	0 [0–0]	0 [0–0]
Virus positive WB_Y	0 [0–0]	0 [0–1]
Virus positive WB_O	0 [0–0]	0 [0–0]
Seropositive WB_M	0 [0–1]	2 [0–3]
Seropositive WB_F	0 [0–1]	1 [0–4]
Seropositive WB_Y	0 [0–2]	3 [0–6]
Seropositive WB_O	0 [0–0]	0 [0–1]
Compliance WB	25 [11–50]	18 [10–27]
FRP presence (yes)	107 (4%)	24 (19%)
FRP culled	85 [15–292]	90 [21–173]
FRP_tested	46 [5–99]	49 [15–195]
FRP virus tested	39 [10–85]	42 [16–187]
FRP sero tested	41 [12–92]	45 [15–194]
Virus positive FRP	0 [0–1]	1 [0–4]
Seropositive FRP	0 [0–32]	18 [2–30]
Sex of the farmer		
Female	37221 (30%)	2009 (25%)
Male	86850 (70%)	6360 (75%)
Age (by 5 years old)	54 [52–57]	55 [49–56]
Educational level (1 = pre-primary, 5 = university)	4 (3,4)	3 (2,3)
Related		
Yes	14888 (12%)	1674 (20%)
Not	109183 (88%)	6695 (80%)
Human population	4957 [1230–10855]	2099 [974–3213]
Q_MDI		
1–very wealthy	623 (22%)	16 (13%)
2– wealthy	410 (14%)	14 (11%)
3–medium	678 (23%)	42 (33%)
4 –deprived	422 (15%)	18 (14%)
5 –very deprived	747 (26%)	37 (29%)
Roads (m^2^)	52,692 [30,660–81,258]	72,333 [53,487–108,929]
Water (km^2^)	24 [3–220]	37 [31–238]
Tourism	0.87 [0.75–1.14]	0.94 [0.91–1.13]
Flood risk population	5.2 ab / km2	6.1 ab / km2
Thefts	16.8 [15.4–25.4]	19.2 [17.5–24.7]
Robberies	0.33 [0.29–0.37]	0.38 [0.37–0.46]
Forest	2795 [1891–7806]	3564 [2411–9952]
Waste	49.5 [46.6–52.1]	44.3 [37.7–49.4]
Energy production	6.8 [6.0–8.2]	6.7 [5.7–7.8]
Roads (m^2^)	52,692 [30,660–81,258]	72,333 [53,487–108,929]
Water (km^2^)	24 [3–220]	37 [31–238]
Employment	52.3 [50.4–52.7]	50.7 [50.5–52.8]

*Data expressed as mean and standard deviation (SD), median and quartile (I–III), frequency (n) and percentage (%), by municipality*.

The random effects u_j_ are M realizations from a multivariate normal distribution with a mean of 0 and q × q variance matrix Σ. The probability that a random response y_ij_ takes the value y and can be modeled by Mixed-effects NBRM is then given by:

(1) $$\begin{array}{l} \mathrm{Pr}\left(y_{ij}=y|{\text{u}}_{j}\right)=\;\frac{\Gamma \left(y+r_{ij}\right)}{\Gamma \left(y+1\right)\Gamma \left(r_{ij}\right)}{p_{ij}}^{r_{ij}}{\left(1-p_{ij}\right)}^{y} \end{array}$$

Univariable NBRM was developed to initially tested each of the explicative variables and to quantify the association between these factors and the distribution of the number of SVDPs. Statistically significant risk factors with *p* ≤ 0.20 in the univariable analysis, were considered for inclusion in the multivariable analysis. Irrelevant risk factors with likelihood ratio test results of *p* ≥ 0.05 were deleted from the multivariable model, based on a stepwise selection procedure (54). Before inclusion into the multivariable model, collinearity presence was evaluated for all those variables with a *p*-value ≤ 0.20 in the univariable analysis, to ensure a variance inflation factor (VIF) <10 (55–57). Interaction therms considered in the multivariable model were between the number of FRPs and ASF-positive WBs. Based on the lowest Akaike information criterion (AIC), if multicollinearity was detected, the predictors involved were identified and one or more was removed (58–61). The final model was assessed using a “training dataset” (years 2015–2018) for internal validation, against a “test dataset” (year 2011–2014) that was not used to create the model, but rather for external validation (62). An assessment of goodness-of-fit of the model between the predicted and observed values was applied, to understand if the data were well-modeled by the NBRM, based on a residuals analysis and the Spearman correlation coefficient. The results of the NBRM are presented in Table 2 as the adjusted odds ratio (ORadj), calculated as proposed by Gardner in 1995 (63). The municipality risk profile was generated, based on values obtained from the NBRM, and the predicted values for each municipality were calculated. To apply EP-ASF15-18 disease control measures, which lay out different actions based on the risk band (from 1 to 5), the predicted values were sorted in ascending order and divided into five equal parts (quintiles, Q1 … Q4). A quintile is one of five values that divide a data range into five equal parts, each being 1/5th (20%) of the range. Given N, the ordered population value (here, the 377 predictor values for each municipality), each quintile is calculated as:

$${\text{Q}}_{\text{j}}=\frac{{\text{j}}^{*}(\text{N}+1)}{5}\text{j}=1,\ldots ,4$$

**Table 2:** Negative binomial regression model results used to obtain the number of ASF positive farms in relation to all known factors related to domestic pigs, wild boars, illegal free-ranging pigs, and farmer sociodemographic characteristics, using data collected in Sardinia 2011–2018.

**Variable**	**OR_adj_ [95% IC]**	***P*-value**
*N* farms	1.013 [1.007–1.025]	< 0.0001
Pigs censed		
< 120	1.00	
≥ 120	2.581 [1.314–5.067]	0.006
Compliance DP	0.821 [0.803–0.867]	< 0.0001
Estimated living WB	1.001 [1.001–1.002]	0.007
Virus positive WB	1.198 [1.042–1.378]	0.011
Virus positive WB_M_perc	1.009 [1.002–1.016]	0.011
Virus positive WB_Y_perc	1.021 [1.001–1.045]	0.039
Sieropositive WB	1.152 [1.049–1.264]	0.003
Seropositive WB_M_perc	1.017 [1.012–1.022]	< 0.0001
Seropositive WB_Y_perc	1.023 [1.015–1.034]	< 0.0001
Compliance WB		
< 21%	1.00	
≥21%	0.604 [0.398–0.916]	0.018
FRP presence	5.067 [3.068–8.368]	< 0.0001
FRP presence * WB positive	1.918 [1.872–1.966]	0.001
Age (by 5 years old)	0.851 [0.740–0.973]	0.019
Sex		
–Female	1.00	
–Male	1.304 [1.176–1.453]	< 0.0001
Human population		
< 5000	1.00	
≥ 5000	0.470 [0.299–0.738]	0.001
Q_MDI		
1–very wealthy	1.00	
2– wealty	1.441 [0.593–3.492]	0.420
3–medium	2.402 [1.173–4.919]	0.017
4 –deprived	1.706 [0.743–3.922]	0.208
5 –very deprived	1.864 [1.385–2.551]	< 0.0001
Roads		
< 70.000	1.00	
≥ 70.000	1.227 [1.031–1.450]	0.023
Employment	0.955 [0.917–0.974]	0.002
Micro-criminality	1.432 [1.418–1.469]	< 0.0001
Tourism	1.196 [1.081–1.325]	0.001
Forest	1.164 [1.038–1.316]	0.013

**Figure 6:**
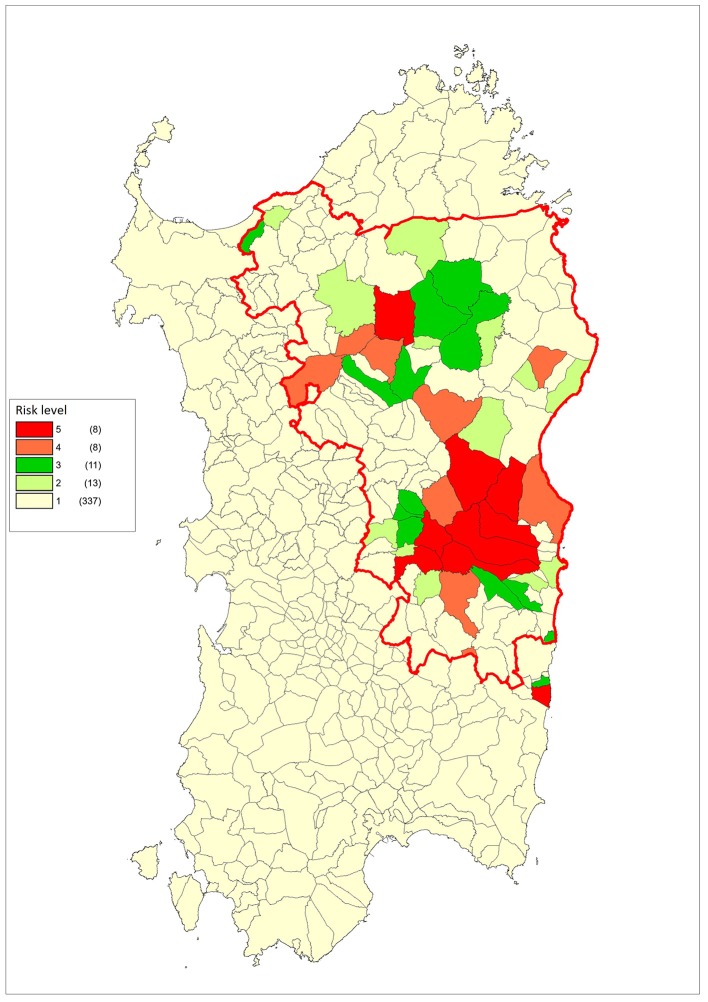
Choropleth map of the different risk levels for each Sardinian municipality.

Figure 6 shows the different risk levels for each Sardinian municipality, and the different type of control measures for each risk band are illustrated in Table 3. All the tests were two-sided and a *p*-value level of 0.05 or less has been considered significant. The statistical analyses were made with R Version 3.3.2 (R Foundation for Statistical Computing, Vienna, Austria) and Stata 13 Release 13 (StataCorp LP, College Station, TX, USA).

**Table 3:** Guidelines for domestic pig farm's control measures, defined by the Sardinian Eradication Plan 2015–2018.

**Risk band**	**Certified farm**	**Checked farm**	**Not-checked farm (during 12 months before)**	**Illegal free-ranging pig breeding**
1–2–3	Clinical check Anagraphical check Biosecurity check Welfare check Serological control **only if** identified risk of disease introduction (*P* ≥ 10%;CI95%)	Follow-up and non-conformities verification: i. Clinical check ii. Anagraphical check iii. Biosecurity check iv. Welfare check Serological control **only if** identified risk of disease introduction (*P* ≥ 10%;CI95%)	i. Clinical check ii. Anagraphical check iii. Biosecurity check iv. Welfare check v. Serologicalcontrol	Contrasting activities to clandestine breeding e illegal handling Including the sanctions/actions of depopulation
4–5	2 × Clinical check 2 × Anagraphical check 2 × Biosecurity check 2 × Welfare check Serologicalcontrol			

## Results

The present risk analysis, conducted to create a risk score, included data related to all Sardinian pig farms, hunting season management, and data from FRP culling actions, based on the 8-year study period (2011–2018). Data were collected by year (*n* = 8) and municipality (*n* = 377). The *ad-hoc* database contained a total 3,016 records. Only 127 records represented municipalities with SVDP cases; all others were equal to a zero value (Figure 5). Descriptive baseline characteristics were divided according to municipalities with zero cases and those with at least one case (Table 1). As expected, most features were different between these two categories. In municipalities with at least one case, factors hypothetically associated with higher risk of ASF spread were more present. Generally, most of the features assessed showed higher levels in municipalities with ASF cases, except for DP and WB compliance, human population, farmer educational level, and employment rate. We tested several potential interactions reported in previous works or according to veterinary experience (i.e., presence of illegal FRPs and WBs, total animal movements and road surface, number of pigs and road surface). However, only the interaction between infected WBs and the presence of FRPs was significant and gave a better-fitting model (AIC: 1126.7 vs. 1171.5). All non-relevant covariates were excluded, as planned in the model approach.

### Negative Binomial Regression Model Results

Overall, 50 variables collected for the period of interest, were considered for inclusion into the final mixed-effects NBRM (Table 1). Totally, 29 were excluded from the final model because of multicollineraity (VIF > 10 and/or Spearman test statistically significant), or owing to a non-significant association with outcome in the univariate analyses (*p* > 0.20). Twenty one of these were finally included, The results obtained by the analysis of the 21 variables are presented in Table 2 and expressed as the ORadj and 95% CI, with *p*-values. The number of farms and pigs in each municipality revealed a role of significant risk factors favoring ASF outbreak, with ORadj 1.013 (95% CI: 1.007–1.025), *p* < 0.001 and ORadj of 2.581 (95% CI: 1.314–5.067), *p* = 0.006 with ≥ 120 pigs on the farm. However, increased farm and veterinary check compliance with the EP-ASF15-18 the previous year significantly decreased the probability of counting one case or more in the same municipality the following year (ORadj = 0.821; 95% CI: 0.803–0.867). The final results of the NBRM showed that a total of eight different features related to the WB population that was live and/or tested positive in the previous hunting season significantly contributed to the risk of ASF cases (*p* < 0.05). In particular, the effect was equal to 1% greater risk (ORadj = 1.001; 95% CI: 1.001–1.002) for each WB estimated to live in the same municipality, and 20% (ORadj = 1.198; 95% CI: 1.042–1.378) and 15% greater risk (ORadj = 1.152; 95% C: 1.049–1.264) if hunted and tested WBs were ASFV positive or ASF-antibody positive, respectively. In addition, an increasing percentage of male WBs that were virus positive and seropositive increased the risk of new SVDPs in the same municipality the following year by 1% (ORadj = 1.009; 95% CI: 1.002–1.016; *p* = 0.011) and 1.7% (ORadj = 1.017; 95% CI: 1.012–1.022; *p* < 0.0001), respectively. Likewise, the probability grew about 2% (ORadj = 1.023; 95% CI: 1.015–1.034; *p* < 0.0001) if positivity (virus or seropositivity) was found in young animals (between age 0 and 6 months). As well as the effect found for DP compliance, compliance with hunting season management rules was a protective factor against the risk of SVDPs the following year. In particular, when WB compliance was greater than 20%, the risk was significantly lowered by 40% (ORadj = 0.604; 95% CI: 0.398–0.916; *p* = 0.018). As hypothesized, the presence of FRPs in the same municipality increased the risk of SVDPs fivefold (ORadj = 5.067; 95% CI: 3.068–8.368; *p* < 0.0001), and about twice if a positive WB was found the previous year in the same municipality as FRPs (ORadj = 1.918; 95% CI: 1.872– 1.966; *p* = 0.001). Older age of farmers seemed to be protective upon an increased number of outbreaks (ORadj = 0.851; 95% CI: 0.740–0.973; *p* = 0.019) whereas the opposite effect was seen for male vs. female sex of the farmer (ORadj = 1.304; 95% CI: 1.176–1.453; *p* < 0.0001). Comparison between the first MDI level (lower deprivation) and others, suggested an increased probability of ASF outbreaks on farms, with statistically significant results between MDI level-1 and MDI level-3 (medium deprivation level) (ORadj = 2.402; 95% CI: 1.173–4.919; *p* = 0.017) or MDI level-5 (very deprived level) (ORadj = 1.864; 95% CI: 1.385–2.551; *p* < 0.0001). Regarding to ISTAT socioeconomic indicators, a low probability of outbreaks on farms located within municipalities with high employment rates (ORadj = 0.955; 95% CI: 0.917–0.974; *p* = 0.002) has been highlighted by the NBRM. Higher counts of outcome variables were observed with higher rates of micro-criminality and tourism in the low season: ORadj = 1.432; 95% CI: 1.418–1.469; *p* < 0.0001 and ORadj = 1.196; 95% CI: 1.081–1.325; *p* = 0.001, respectively. Finally, regions with asphalted road area of more than 70,000 m^2^, high forest surface coverage, and a human population of <5000 people showed a significantly higher risk of SVDPs (*p* < 0.05). The results of the likelihood ratio test (LR, *X*^2^ = 262.55, probability > *X*^2^ = 0.0001) supported the choice of the mixed-effects NBRM against the mixed-effects Poisson regression model. Based on internal validation criteria (residual mean = 4.18^*^10^−5^, SD = 2.05^*^10^−6^, Spearman correlation coefficient = 0.846, *p* < 0.0001) and external validation criteria (residual mean = 3.99^*^10^−3^, SD = 7.82^*^10^−4^, Spearman correlation coefficient = 0.793, *p* < 0.0001), it is possibile to affirm that the NBRM could properly predict the correct outcome with a strong goodness-of-fit.

## Discussion

Sardinia is the European area that has been affected by ASF the longest, since its first notification in 1978. Furthermore, Sardinian territory is the only region “where the epidemiological situation has become stabilized and the disease has become endemic,” such that the region is the only one on the European continent included in Part IV (highest risk) of the European Commission Decision on ASF control measures (Decision 2014/709/EU). Despite the rapid spread of ASFV across Europe and parts of the Asian continent, excluding an isolated and quickly resolved case in northern Italy in 1983 owing to illegal introduction of pork from Sardinia, there is no evidence of disease spread from Sardinia to other countries (6). As demonstrated by many studies on the Sardinian ASFV genotype, Sardinian isolates are included in a cluster of genotype I (64–66), whereas genotype II circulates in other European countries, Transcaucasia, Russia, and China (67). This very low genetic variability determines the placement of strains into one of two clusters depending on the temporal distribution: subgroup III, including viruses isolated up to 1990, and subgroup X, including isolates identified from 1990 to 2009 (68, 69) A total of 11 outbreaks occurred during the first 8 months of last year whereas from September 2018 to the present, no virus evidence has been reported on DP farms in Sardinia. Nevertheless, in the previous hunting season (1 November 2018 to 31 January 2019), a total of four WBs were found to be ASFV positive and 106 presented antibodies against ASF. The prevalence of the disease during the past 7 years has decreased drastically in Sardinia, among both wild and domestic populations. During the 40 years of control and eradication efforts against ASF, different regionaleradication plans have been implemented, many of which are similar to those applied in countries where the disease has been eradicated, such as Spain and Portugal (70, 71) and some countries have been able to almost entirely eradicate ASF. However, the last eradication plan in Sardinia achieved the most striking results in terms of significant decline in disease among all the suid populations involved. The EP-ASF15-18 addresses not only improved target veterinary measures but also measures to eliminate FRPs to better manage the hunting season and animal movements, and providing greater general incentives toward good biosecurity practices. In particular, this plan is focused on checks and measures to be applied on DP farms, planned by year. As reported in Table 3, different timetables are planned based on municipality risk level. Until now, classification of each municipality's risk level was performed using qualitative analysis (39). Ours is the first work to describe the risk level based on the results of multivariable predictors, with external and internal data validation. From numerous previous studies as well as endemicity of the disease for more than 40 years, it is now known that the situation of ASF in Sardinia is very different from that of all other countries. It is almost as if the virus has found its perfect conditions for thriving within the unique Sardinian epidemiological cycle (6, 13, 14, 17, 18, 27, 31, 43, 72, 73). As described by Laddomada et al., in 2019 (18), the strong measures applied against illegal FRP populations have marked a turning point in the story of the fight against ASF in Sardinia, with record results in terms of declines in the disease. The typically Sardinian epidemiological cycle, described earlier in the present work, involves the three Sardinian suid populations, generating a virus transmission cycle that is very difficult to control, given the role of FRPs as a link between the WB population and DPs. For these reasons, the quantitative risk analyses performed here has taken into account many different features related not only to pigs bred in backyard farms, but also the local WB population and pigs that are illegally bred in a free-range manner, as well as the role of socioeconomic and demographic factors. Some results found in previous studies have been confirmed in this work for DP farms, such as the contribution of the number of farms and recorded number of animals to new ASF outbreaks in the same municipality (17, 27, 43). As demonstrated by the FAO (26), the key roles of both animal density and low biosecurity in disease maintenance are evident. The results of our study underline this relationship, showing a statistically significant increased risk with increased DP population and WB density, as well as the presence of the third population (FRPs). Furthermore, the simultaneous presence of FRPs and infected WBs doubles the risk of observing ASF infections on farms the following year. Thus, the close coexistence of domestic and wild pig species makes disease management more difficult, as underlined by Pastoret et al. (74). The problems related to this situation are many and complex because the geographical, ecologic, and economic conditions that permit transmission among populations are different and extremely variable, as is surveillance. Whereas, the situation may be relatively simple for domestic animals, the same consideration may not be applied to wild species, given the differences in their variety and population density. The Sardinian situation is complicated even more by related social and cultural issues that hinder ASF eradication. First is the cultural identity of pig farmers and resistance to respecting control measures, particularly those regarding elimination of FRPs, an ancient practice that is culturally rooted in the central Sardinian region (6, 17, 18, 38, 72, 73). The Sardinian context is that of small communes with very few inhabitants and almost no services that follow ancient and time-honored cultural traditions, in contrast with larger cities with very crowded areas and a capital defined as a metropolis. The need to take into account socioeconomic status has been suggested by the World Organization for Animal Health Guidelines in 2014, which have affirmed that animal disease management should consider several non-financial factors (i.e., social, cultural, and religious) affecting the livelihood and well-being of animal owners such as pastoralists, farmers, and small-scale backyard producers. These factors can be important incentives in participation or non-compliance and can ultimately impact the success of sanitary programmes (75). In Sardinia, the need to include social and economic factors in risk analysis is particularly pertinent, since animal farming has always been one of the main economic resources. With reference to these particular Sardinian conditions, according to expert opinions and previous studies, the greatest risk for ASF spread and persistence has been determined to be located in smaller countries and rural contexts (17, 32). All social features included in the present work contribute to describing the typical Sardinian situation where high-risk areas are identified in deprived municipalities (Q_MDI = 5) with very few inhabitants, low employment rates, and high levels of micro-criminality. Furthermore, farmers at high risk of being associated with SVDPs were found to be young males with low educational levels, as also reported by Loi et al. for many different diseases in Sardinia (32). Although these factors are not directly associated with ASF development and spread, they could help to create conditions under which the disease can spread. Numerous limitations of the present work are related to data traceability, accuracy, and underreporting data. However, the checks carried out before at the beginning of the analysis may have been at least partly limited by problems related to registration in the BDN, leading to possible generation of selection bias. Furthermore, the present study is not exempt from the typical limitations of risk analysis with the use of proxies, which may give a reflected measure, characterized by evident less accuracy, of features not directly measured. For example, the significant role of tourism in the low season as an indicator of disease occurrence should not be interpreted as a direct effect but rather as a proxy for a low biosecurity context. During the previous autumn and winter seasons in particular, different traditional popular festivals take place one after the other in central Sardinia, during which pig meat products are elaborated and sold, sometimes without permission in an illegal context and without veterinary controls, favoring contamination by and spread of ASFV. These events are typical of inland areas, where a higher number of farms are recorded and where the epicenter of the disease has been identified in many studies conducted over the last 40 years (6, 13, 18, 31, 71, 76, 77). Although the results of the present work were obtained using data of Sardinia and are specific to this context, and despite the use of specific social variables using an Italian database (ISTAT), our findings can be considered a point of departure for future investigation. Furthermore, the present risk analysis reveals many new and unique details regarding the Sardinian ASF cycle (i.e., the interaction between infected WBs and FRPs and their association with ASF risk on DP farms, and the valid and effective use of social factors to describe at-risk areas). Further confirmation of our results, together with previous knowledge about this disease, could be useful to understanding the disease cycle in countries with similar conditions such as Ukraine and other parts of Eastern Europe (78). The implementation of the latest eradication plan and its effectiveness throughout Sardinian territory (described by the two compliance measures) contributed to the large observed decrease in ASF during the past 6 years, although the region remains endemic. As outlined previously, active surveillance conducted in endemic areas with decreasing prevalence is generally the most suitable approach, which includes monitoring the effect of interventions on the prevalence of infected animals. However, the EFSA's suggestions for countries where the disease is endemic in WB, such as Sardinia, define the passive surveillance as the most effective and efficient method for early detection of ASF in WBs, particularly in areas where ASF has not been detected for several time (79, 80). The decrease of serological and virological findings, indicating levels of disease activity, and accompanying improvement of the situation in DPs and WBs suggest the need to continue this strategy through the final phase of the eradication program.

## Data Availability

All datasets generated for this study are included in the manuscript/Supplementary Files.

## Author Contributions

FL, SC, AC, and SR: conceptualization, investigation, writing—original draft, review, and editing. FL: data curation, formal analysis, and validation. FL and SR: methodology. SR and AC: project administration, resources, and supervision. SC and FL: visualization.

### Conflict of Interest Statement

The authors declare that the research was conducted in the absence of any commercial or financial relationships that could be construed as a potential conflict of interest.
