# Supplementary material for: Standardized Risk Analysis Approach Aimed to Evaluate the Last African Swine Fever Eradication Program Performance, in Sardinia
Source: Front Vet Sci. 2019 Sep 13;6:299. doi: 10.3389/fvets.2019.00299 (PMC6753231; doi:10.3389/fvets.2019.00299)
Supplement: Supplementary file 1 [file Table_1.DOCX]

**S1 Table.** List of variables included in the retrospective analyses to evaluate the specific municipality-level risk of African swine fever persistence or reoccurrence in Sardinia, Italy during 2011–2018,

according to five categories including domestic pigs, wild boards, illegal free-ranging pigs, and socioeconomic factors.

| **Variables** | **Description** | **Abbreviation** | **Category^*^** |
| --- | --- | --- | --- |
| y | Number of seropositive and virus positive domestic pigs | SVDP | A |
| x1 | Year of data collection | Year | A, B, C, D, E |
| x2 | Local Socio-Sanitary Agency of reference | ASSL | A, B, C, D, E |
| x3 | Municipality of reference | Municipality | A, B, C, D, E |
| x4 | Infected or Not Infected zone | ZI – NZI | B§ |
| x5 | Number of farms censed (at 31 December) | N farms | A |
| x6 | Total number of pigs censed | Pigs censed | A |
| x7 | Number of seropositive domestic pig farms | Seropositive farms | A |
| x8 | Number of virus positive domestic pig farms | Virus positive farms | A |
| x9 | Number of veterinarians official checks in pig farms | Farms checked | A |
| x10 | Data about animal movements (number of animals  introduced/leaving farm from municipality to municipality) | Movements | A |
| x11 | Percentage of Compliance in domestic pigs, defined as the | Compliance DP | A |
| x12 | Areal of WB | Areal | B§ |
| x13 | Number of estimated WB living | Estimate living WB | B§ |
| x14 | Number of estimated WB to be hunted (45% of total number  of estimated WB living) | Estimate hunted WB | B§ |
| x15 | Number of WB hunted | Hunted WB | B§ |
| x16 | Sex of WB hunted | Sex WB | B |
| x17 | Age of WB hunted | Age WB | B§ |
| x18 | Number of WB tested for ASFV presence | WB virus tested | B§ |
| x19 | Number of WB tested for ASF antibodies presence | WB sero tested | B§ |
| x20 | Number of WB ASF virus positive | Virus positive WB | B§ |
| x21 | Number of WB ASF seropositive | Seropositive WB | B§ |
| x22 | Number of male WB ASF virus positive | Virus positive WB_M | B§ |
| x23 | Number of female WB ASF virus positive | Virus positive WB_F | B§ |
| x24 | Percentage of male WB ASFV positive, over all WB ASFV  positive | Virus positive  WB_M_perc | B§ |
| x25 | Number of young WB ASF virus positive (0 - 6 months) | Virus positive WB_Y | B§ |
| x26 | Number of young WB ASF virus positive (6 - 30 months) | Virus positive WB_O | B§ |
| x27 | Percentage of young WB ASFV positive, over all WB  ASFV positive | Virus positive WB_Y_perc | B§ |
| x28 | Number of male WB ASF seropositive | Seropositive WB_M | B§ |
| x29 | Number of female WB ASF seropositive | Seropositive WB_F | B§ |
| x30 | Percentage of male WB ASF seropositive, over all WB ASF  seropositive | Seropositive WB_M_perc | B§ |
| x31 | Number of young WB ASF seropositive (0 - 6 months) | Seropositive WB_Y | B§ |
| x32 | Number of young WB ASF seropositive (6 - 30 months) | Seropositive WB_O | B§ |
| x33 | Percentage of young WB ASF seropositive, over all WB  ASF seropositive | Seropositive WB_Y_perc | B§ |
| x34 | Total number of WB tested positive for ASFV or ASF  antibodies presence | WB positive | B§ |
| x35 | Percentage of Compliance in WB, defined as the proportion  of WB hunted over the total WB estimated to be hunted in the same municipality, during the year of reference | Compliance WB | B§ |
| x36 | Presence/absence of illegal free-ranging pigs | FRP presence | C |
| x37 | Number of illegal free-ranging pigs culled | FRP culled | C |
| x38 | Number of illegal free-ranging pigs tested for ASFV  presence | FRP virus tested | C |
| x39 | Number of illegal free-ranging pigs tested for ASF  antibodies presence | FRP sero tested | C |
| x40 | Number of illegal free-ranging pigs ASF virus positive | Virus positive FRP | C |
| x41 | Number of illegal free-ranging pigs ASF seropositive | Seropositive FRP | C |
| x42 | Sex of the farmer (Female/Male) | Sex | D |
| x43 | Age of the farmer | Age | D |
| x44 | Educational level (1 to 5) | Educational level | D |
| x45 | Relationship with other farms within 2 km (Yes/Not) | Related | D |
| x46 | Human population (total number of people) | Human population | D |
| x47 | Quintiles of Material Deprivation Index (MDI) | Q - MDI | D |
| x48 | Asphalted roads area (m2) | Roads | D |
| x49 | Water bodies area (km2) | Water | D |
| x50 | Employment rate | Employment | D |
| x51 | Cultural demand | Culture | D |
| x52 | Micro criminality index | Micro-criminality | D |
| x53 | Rate of tourism in not-summer period | Tourism | D |
| x54 | Amount of flood risk population | Flood risk population | D |
| x_55_ | Rate of reported thefts | Thefts | D |
| x_56_ | Rate of reported robberies | Robberies | D |
| x_57_ | Forest surface | Forest | D |
| x_58_ | Municipal differentiated waste | Waste | D |
| x_59_ | Energy produced from renewable sources | Energy production | D |

*Categories concerned data related to domestic pig farms (category A), wild boar (category B), illegal free-ranging pigs (category C), and socio-economic characteristics (category D)

^§^all features of B category collected have been taken in relation to previous hunting season respect the year of collection of the domestic pig farms characteristic
